# Vibration behavior of diamondene nano-ribbon passivated by hydrogen

**DOI:** 10.1038/s41598-019-52343-x

**Published:** 2019-10-31

**Authors:** Lei Wang, Ranran Zhang, Jiao Shi, Kun Cai

**Affiliations:** 1 Department of Engineering Mechanics, College of Mechanics and Materials, Hohai University, Nanjing, 211100 China; 2 College of Water Resources and Architectural Engineering, Northwest A&F University, Yangling, 712100 China; 3 State Key Laboratory of Structural Analysis for Industrial Equipment, Dalian University of Technology, Dalian, 116024 China; 4 Centre for Innovative Structures and Materials, School of Engineering, RMIT University, Melbourne, VIC 3001 Australia

**Keywords:** Chemical physics, Nanoscience and technology

## Abstract

Diamondene is a new kind of two dimensional carbon allotrope with excellent properties and passivation approaches are often used to reduce the extremely high pressure required during its fabrication. When a one-end-clamped diamondene ribbon is hydrogenated on one surface, the ribbon tends to bend and vibrate due to asymmetric layout of C-H bonds on two surfaces. In the present work, the vibration behavior, including natural curvatures and vibration frequencies of diamondene ribbons, were investigated by molecular dynamics simulations. Results indicate that the natural curvature radius of a narrow diamondene ribbon is close to 12.17 nm at a temperature below 150 K, which is essential for fabricating an arc nanodevice. The first order frequency (*f*_1_) of a cantilever beam made from the ribbon follows traditional beam vibration theory if the slenderness ratio is low. In particular, *f*_1_ increases logarithmically at temperature below 50 K, but changes slightly between 50 K and 150 K. It suggests a design scheme for a nanoresonator with temperature-controlled frequency.

## Introduction

With specific electron configurations, from zero-dimensional fullerene to 3-dimensional diamond or lonsdaleite, carbon allotropes are well-known for their physical properties. Especially, graphene is single-layered graphite. As one of the most popular two-dimensional materials, it has attracted much attention since obtained in laboratory in 2004^1^, and has been used widely^2–4^.

A graphene sheet has perfect in-plane modulus, strength^5,6^, thermal conductivity^7,8^, and electron mobility^9,10^. Due to existence of *p*-electrons, the inter-layer interaction between neighbor graphene sheets is determined by non-bonding/van der Waals force. Correspondingly, the extremely low inter-layer friction of adjacent sheets benefits dynamics devices, e.g., graphene-based nano-motor^11–13^. Moreover, few-layered graphene sheets behave orthogonal thermal conductivity, i.e., the out-of-plane conductivity is far less than that of in-plane property^14^. Once a material with high shear strength and isotropic thermal conductance is required as a cover in a nano-device, graphene sheets do not meet such requirement. Considering isotropic thermal and mechanical properties of diamond, people desire for developing a two-dimensional material called diamond film^15–20^, which behaves nice thermal and mechanical properties in the third dimension. Phase transition from sp^2^ to sp^2^/sp^3^ hybridization of carbon material was discovered for over half a century^15^, but recently observed via Raman spectrum^21^ in compressing few-layered graphene sheets. The new carbon material, which is called diamondene/diamene, was also estimated by first-principles calculation, as well^18^. Soon after that, the thermal stability and mechanical properties of either diamondene ribbons or nanotube were investigated^22–24^.

In forming diamondene from graphene sheets, the extremely high pressure together with high temperature is a key challenge but essential for phase transition. The reason is that *p*-electrons are anti-bonding electrons, which are difficult to form a covalent bond at ambient conditions of either low pressure or low temperature. To deal with this difficulty, the surfaces of graphene sheet were passivated by either hydrogenation^19,20^ or bonding with other materials^25^. Single-sided hydrogenation on graphene can lead to specified bending angles and thus provide a method to precisely manipulate graphene into complex structures^26^ and its morphology can furthermore be tuned via an external electric field^27^. For a diamondene ribbon with one hydrogenated surface, it can become a ribbon with only one side containing C–H bonds for potential application. If so, the diamondene ribbon will curve toward the non-hydrogenated side along both in-plane dimensions due to asymmetric layout of C–H bonds in two surfaces. Similarly, asymmetry can also be utilized by precursor films to cause bending and unwrapping of graphene^28^. The naturally curved material can be used in fabricating an arc device, e.g., stators in a nano-universal joint^29^ or curved resonators for obtaining beat vibration^30^. In this study, the primary physical properties, e.g., the natural curvature and natural vibration frequency of such diamondene ribbons, were investigated by molecular dynamics (MD) simulations, and possible applications were predicted based on these results.

## Model and Methodology

**Figure 1:**
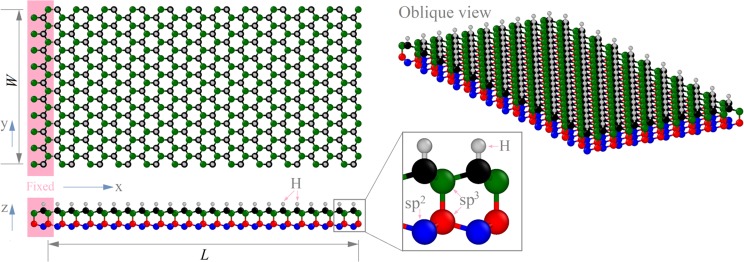
Schematic of cantilever nanobeam from a diamondene ribbon with sizes of *L* × *W*. The top carbon atoms (in black) in the ribbon are hydrogenated. In the ribbon, only the bottom carbon atoms (in blue) are sp^2^ atoms. *L* varies from 9.6 nm to 29.6 nm, and *W* is between 1.6 nm and 7.3 nm. The left end of the ribbon is fixed and the right end is free.

In Fig. 1, a diamondene ribbon with the upper surface being hydrogenated is fixed at its left end. The right end is free. The length and width of the ribbon are labeled as *L* and *W*, respectively. Due to the existence of the C–H bonds on the upper surface, the nanobeam will bend downwardly in relaxation. The effects of temperature (T) between 0.1 K and 150 K, 9.6 nm < *L* < 29.6 nm, and 1.6 nm < *W* < 7.3 nm, on the curvature and natural vibration frequency were considered in this study. The MD simulations were carried out in the open source code LAMMPS^31^. The interaction between atoms in the hydrocarbon system was evaluated by the AIREBO potential^32^, which has been widely used in MD simulations. In each simulation, the ribbon was first built. Before relaxation, the system was reshaped by minimizing its potential energy. After that, the system in a large box with periodic boundary conditions along three dimensions was relaxed in a NPT ensemble. During relaxation, the atoms close to the left end are kept to be fixed. In time integration, the time increment was set to be 0.2 fs. Within each 1000 steps, the simulation results were averaged and recorded for post-processing.

To show the natural vibration, more than one period of vibration of the cantilevered nanobeam was simulated. The natural frequencies were obtained by fast Fourier transition (FFT) approach^33,34^. To describe the deformation of the ribbon, both configurations and the variation of potential energy (VPE) were provided. Mathematically, VPE can be defined as

1 $${\rm{VPE}}={\rm{PE}}(t)-{\rm{PE}}({t}_{0})$$

where PE(*t*) and PE(*t*_0_) represent the current and initial potential energy of the system, respectively. They can be obtained by substituting the locations of atoms in the system into AIREBO potential function. On the other hand, PE can be considered as a summation of two items. One is the potential energy of the system in the stable state, i.e., the system contains only kinetic energy with respect to thermal vibration, which is labeled as TPE. The other is the potential energy caused by deformation of the cantilevered beam from its stable state, i.e., labeled as DPE. At a specific temperature, TPE is constant, and therefore, the value of VPE can be expressed as the difference between the current and initial values of DPE, i.e.,

2 $${\rm{VPE}}={\rm{DPE}}(t)-{\rm{DPE}}({t}_{0}).$$

Once *t*_0_ is chosen at the moment of the system in the stable state, DPE(*t*_0_) = 0, and DPE(*t*) > = 0. It means that the deformation of the system from its stable state will result in increase of potential energy.

## Results and Discussion

**Figure 2:**
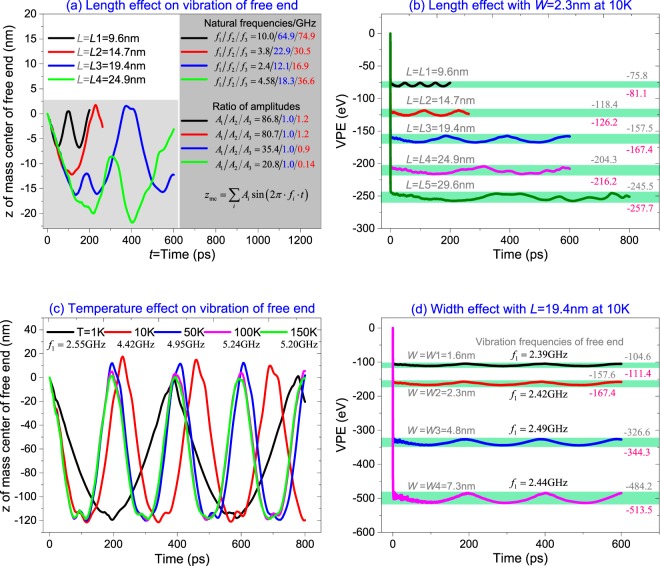
Parameter study on natural vibration of diamondene ribbons. (**a**) Vibration curves of the free end and (**b**) VPE of ribbons with different lengths but the same width of *W* = 2.3 nm at 10 K; (**c**) Vibration curves of the free end of the ribbon with sizes of 14.7 nm × 2.3 nm at different temperatures; (**d**) VPE curves of the ribbons with different width but the same length of *L* = 19.4 nm at 10 K, and the first order frequency of the free end’s vibration was labeled.

The natural vibration of the free end of the diamondene ribbon was illustrated by the historical curve of its mass center on z direction, which was shown in Fig. 2a. It can be found that the amplitude of z(*t*) increases with the length of the ribbon. By using sine-FFT algorithm, the first three orders of natural frequencies and the related amplitudes were obtained for each case of ribbon length. Results show that the first order frequency (i.e., *f*_1_) is about 10 GHz for the cantilevered diamondene beam with span of *L* = *L*1 = 9.6 nm. When the span is increasing, e.g., *L* = *L*2 = 14.7 nm or *L* = *L*3 = 19.4 nm, the value of *f*_1_ decreases. According to the traditional beam vibration theory, the relationship between *f*_1_ and span of a cantilevered beam satisfies ${f}_{1}\propto {L}^{-2}\sqrt{EI/\rho A}$, where *EI* being bending stiffness, *ρ* as material mass density and *A* as cross-sectional area of beam, respectively. We found that this rule is obeyed when *L* < 20 nm. When *L* = *L*4 = 24.9 nm, *f*_1_ is higher than that of the beam with *L* = *L*2. The reason is that the free end of the beam with *L* = *L*4 mostly vibrates about a cross-section of beam which is away from the fixed end. Theoretically, for a slimmer ribbon (L/W is higher), it contains more vibration modes, and the higher order modes become stronger.

**Figure 3:**
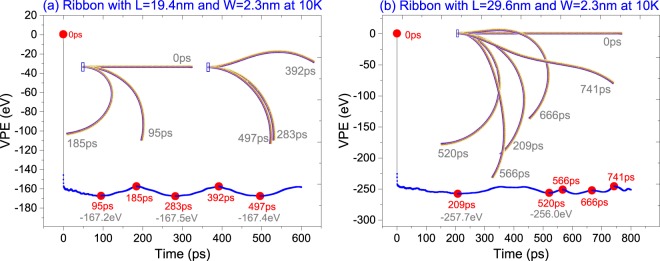
Configurations of the ribbons during relaxation at 10 K. (**a**) The beam with sizes of 19.4 nm × 2.3 nm. (**b**) The 29.6 nm × 2.3 nm diamondene beam.

By comparing the second and third orders of frequencies (i.e., *f*_2_ and *f*_3_), we also found that the magnitude of *f*_2_ or *f*_3_ decreases when *L* increases from 9.6 nm to 19.4 nm. It becomes higher at *L* = *L*4 = 24.9 nm. When comparing the amplitudes (*A*_i_) related to the three orders of frequencies, we found that the contribution of the higher-order vibrations of the diamondene beam is much lower than that of the first order when *L* = *L*1 or *L*2. When *L* = *L*3, the contribution of the higher order vibrations becomes larger. For example, *A*_1_/*A*_2_/*A*_3_ = 86.8/1.0/1.2 when *L* = *L*1, or 80.7/1.0/1.2 when *L* = *L*2. The ratios become 35.4/1.0/0.9 when *L* = *L*3 or 20.8/1.0/0.14 when *L* = *L*4. Hence, the contribution of the second order vibration becomes stronger when the span of beam increases. In particular, for the beam with *L* = *L*4, the configuration of the beam is mainly determined by the first and the second order modes. The detailed dynamic behavior will be addressed by the results in Fig. 3.

In Fig. 2b, the evolution of the potential energy of the diamondene ribbon was shown. For a beam, its potential energy decreases steeply within the first 0.4 ps, which is mainly caused by the adjustment of both bond lengths and bond angles in the ribbon from their initial uniformly distributed state to a stable state. During the very short period, the configuration of the beam is almost unchanged, i.e., the ribbon still looks like a plate. But later, the VPE curve fluctuates within a constant interval, e.g., −81.1 eV < VPE < −75.8 eV when *L* = *L*1. As aforementioned, the beam is in the stable state when VPE reaches the minimum. If it deforms further, no matter bending further or becoming flat, VPE will be higher (Eq. (2)). And the two neighbor peaks on the VPE curve give two extreme deformations of the diamondene beam.

It can also be found that the curves with respect to *L* < 20 nm fluctuate periodically, and the period is about half of that for z(*t*). The two characteristics demonstrate that the ribbon with *L* < 20 nm and *W* = 2.3 nm is in the first order vibration mode. This can be verified by the results in Fig. 3a. However, if the beam has a larger span, its vibration will be determined by both the first and the second orders of vibration mode, which can be demonstrated by the snapshots in Fig. 3b.

Figure 2c show the temperature effect on the vibration of a beam with specific span, e.g., *L* = 14.7 nm. If temperature is different from 10 K, the first order frequency is different, as well. In particular, between 1 K and 50 K, *f*_1_ increases logarithmically with temperature. But between 50 K and 150 K, the frequency fluctuates slightly. Hence, the diamondene ribbon is softer at lower temperature (if T < 50 K). Between 100 K and 150 K, the ribbon has a constant bending stiffness, i.e., the stiffness is independent of temperature. The two properties can be adopted for design of a temperature-dependent nano-device, e.g., nano-resonator^30,35,36^.

In Fig. 2d, VPE curves of the diamondene ribbons with the same span but different widths were given, and the first order vibration frequencies of their free ends at 10 K were also labeled nearby. One can find that the amplitudes of fluctuation of the VPE curves increase linearly with the widths, but the first order frequencies are different slightly, e.g., *f*_1_ = 2.39 GHz at *W* = *W*1 = 1.6 nm, or *f*_1_ = 2.49 GHz at *W* = *W*3 = 4.8 nm, or *f*_1_ = 2.44 GHz at *W* = *W*4 = 7.3 nm. It implies that the width of the diamondene beam (*W* ≤ 7.3 nm vs. *L* = 19.4 nm) slightly influences the bending stiffness of the beam, i.e., the hydrogenation of top surface leads to slight curvature in the z-direction.

To illustrate the VPE value and the configuration of ribbon during vibration, two diamondene ribbons were considered here. Figure 3a gives the VPE curve of the diamondene ribbon with sizes of 19.4 nm × 2.3 nm. Within the first 500 ps, VPE has three local minima at 95 ps, 283 ps and 497 ps, respectively. By comparison, the VPE at 95 ps is the lowest in this period. By observing the snapshot at 95 ps, we found that the bended diamondene ribbon has nearly uniform curvature. Hence, we can estimate the radius of the ribbon at the moment. And this radius is the natural curvature radius of diamondene ribbon with only one surface being hydrogenated.

In Fig. 3a, we also found that the snapshots at 283 ps and 497 ps were slightly different from that at 95 ps. It means that the three snapshots are close to the really stable configuration of the ribbon after full relaxation. At 185 ps and 392 ps, the configurations were different obviously, e.g., the beam was over bended at 185 ps, and became flat at 392 ps. Hence, as aforementioned, the two configurations have approximate VPE values despite their much different bending states. As comparing all of the configurations in Fig. 3a, we also find that the beam was in the first order mode when neglecting the front part of the beam (which changes slightly during vibration).

In Fig. 3b, some representative snapshots of the diamondene beam with sizes of 29.6 nm × 2.3 nm were given according to the related VPE curve. At 520 ps, the beam has the minimal VPE, and almost uniform curvature. At other moments, the snapshots are different from the stable configuration. At 209 ps, even if the VPE value is slightly different from the minimum at 520 ps, the position of the beam is much different from that at 520 ps. The reason is that the main front part of the beam is rotating about the part which is close to the fixed end. At 566 ps, 666 ps, and 741 ps, the VPE values are at local maxima. But the beam at 666 ps is over bended, while at 566 ps or 741 ps, the beam becomes flat. In particular, the configuration at 741 ps indicates the contribution of higher order mode of vibration.

**Figure 4:**
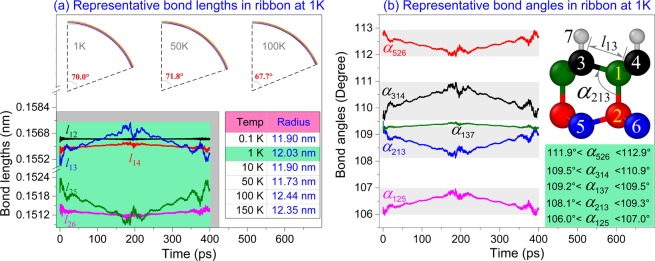
Representative bond lengths and bond angles in the ribbon (14.7 nm × 2.3 nm) relaxed at 1 K. (**a**) Representative bond lengths. Curvature radius of the ribbon at different temperatures were also listed. (**b**) Representative bond angles at 1 K.

In context, the method for finding the stable configuration of a beam has been suggested, and accordingly, the curvature radius can be calculated based on the span and the radius angle. For the diamondene beam with sizes of 14.7 nm × 2.3 nm, the radius is between 11.90 nm and 12.44 nm at temperature between 0.1 K and 150 K (see the Table inset in Fig. 4a). Hence, the curvature of a slim diamondene ribbon with only one hydrogenated surface is almost independent of temperature. This property is essential for fabricating an arc device on the nanoscale.

To demonstrate the mechanism of bending of the beam in vibration, the representative bond lengths and bond angles at 1 K were given in Fig. 4. Bending of the ribbon is caused by the asymmetric geometry of the cells in the ribbon. Bending curvature can be expressed as the deflection of the centroid of a cell (e.g., inserted in Fig. 4b) with respect to the span (the length in x-direction). If angle *α*_213_ = *α*_125_, the centroid has no deflection. If *α*_213_ > *α*_125_, the centroid moves downward. Bending of the “cross section” of the cantilevered beam is generated mainly by the difference between the two angles.

According to the vibration curve in Fig. 2c, the ribbon completes a period of vibration within 400 ps. It can be found in Fig. 4a, bond length *l*_13_ increases faster in the first 200 ps, and then decreases. Oppositely, *l*_25_ decreases first and then increases. The other bond lengths change slightly in this period. We also know that in the first 200 ps, the diamondene ribbon bends downward. Hence, bonds 1–3 and 1–4 is under tension while bonds 2–5 and 2–6 under compression. But the intersection angle between bond 1–3 (or 2–5) and x-axis is lower than that between bond 1–4 (or 2–6). Hence, bond 1–3 varies faster than bond 1–4 in bending. This can also be verified by the change of the bond angles in Fig. 4b. For example, in the first 200 ps, *α*_314_ and *α*_125_ increase, while *α*_526_ and *α*_213_ decrease. The differences among the initial value of the representative bond angles are determined by the hydrogenation scheme, e.g., atoms 3 and 4 are sp3 atoms after hydrogenation, while atoms 5 and 6 are still sp2 atoms. Hence, *l*_13_ > *l*_25_, but *α*_526_ > *α*_314_.

## Conclusions

In experiments, diamondene can be obtained by putting two or more layers of graphene under extremely high pressure at room temperature. To reduce the external pressure and to obtain a diamond film with high thermal stability, in general, the surface layers are passivated by hydrogen. Once only one surface of a diamondene ribbon is hydrogenated, the free ribbon bends due to asymmetric layout of C-H bonds in two surfaces. To show the curvature and the stiffness of the ribbon, in this study, a cantilevered nanobeam made from a narrow rectangular diamondene ribbon was studied by MD simulations. Results indicate some essential conclusions for potential application of such new low-dimensional material, i.e.,

1. The curvature radius of a narrow diamondene ribbon is ~12.17 nm ± 0.27 nm at temperature below 150 K. It implies that curvature is not sensitive to temperature. This characteristic is significant for fabricating an arc nano-device.
2. For a diamondene ribbon based nanobeam with low slenderness ratio, e.g., span *L* < 20 nm vs. width *W* = 2.3 nm, its first order vibration frequency, i.e., *f*_1_, obeys ${f}_{1}\propto {L}^{-2}\sqrt{EI/\rho A}$ with *EI* being bending stiffness, *ρ* as material mass density and *A* as cross-sectional area of beam, respectively.
3. The value of *f*_1_ increases logarithmically at temperature below 50 K. But between 50 K and 150 K, it changes slightly. Hence, a nano-resonator with temperature-controlled frequency can be designed.
4. For a diamondene beam with span less than 20 nm, *f*_1_ changes slightly when the width is less than 7.3 nm.

## Data Availability

The raw/processed data required to reproduce these findings cannot be shared at this time as the data also forms part of an ongoing study.
